# Supplementary material for: Transcriptomes and neurotransmitter profiles of classes of gustatory and somatosensory neurons in the geniculate ganglion
Source: Nat Commun. 2017 Oct 2;8:760. doi: 10.1038/s41467-017-01095-1 (PMC5624912; doi:10.1038/s41467-017-01095-1)
Supplement: Supplementary file 1 — Supplementary Information [file 41467_2017_1095_MOESM1_ESM.pdf]

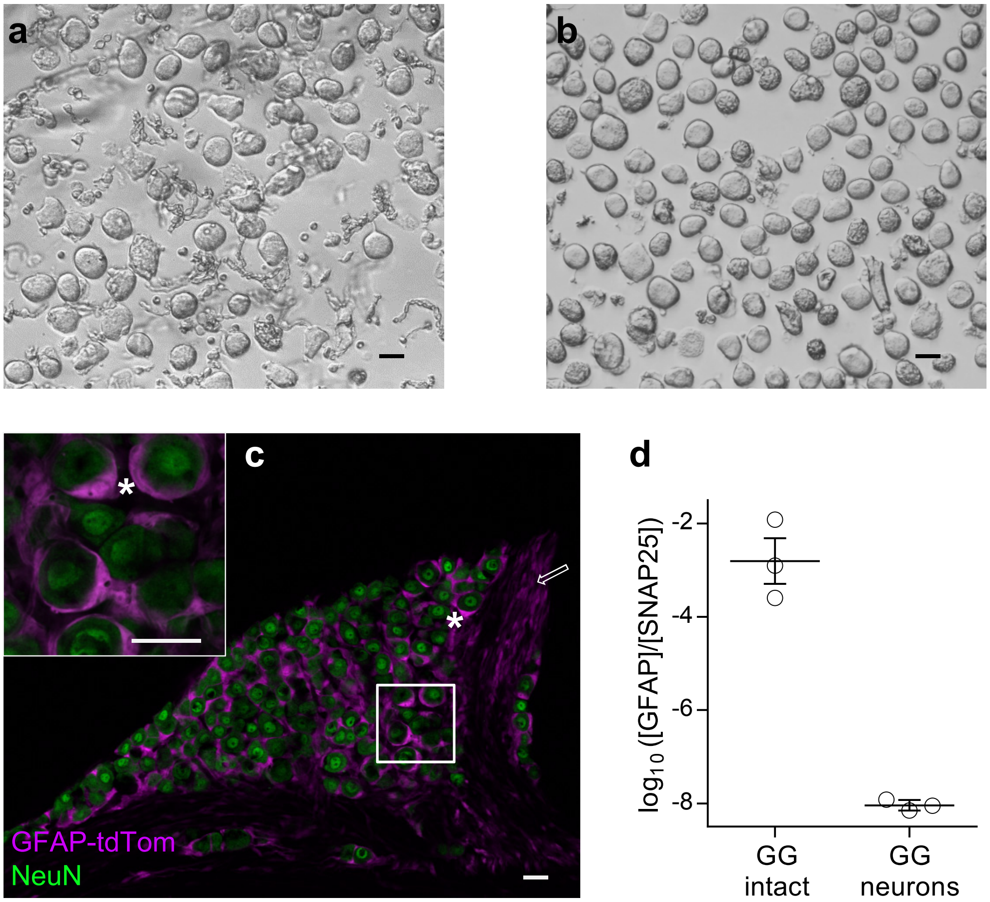

Supplementary Figure 1. Dissociation and purification of geniculate ganglion neurons. a. Dissociated geniculate ganglion preparations include neurons, satellite glia, and axon fragments. **b.** After manual purification (see Methods), the preparation is more homogeneous and contains mostly neurons. **c.** Confocal micrograph of a cryosection of geniculate ganglion from a *Gfap*;tdTom mouse, showing that many satellite glia (★) and Schwann cells (➚) in the nerve express tdTomato (pseudocolored magenta), and by inference, GFAP. Neurons are visualized by immunostaining for NeuN (green). Note that some satellite glia are clearly GFAP+ while others are negative. Whether this reflects molecular and functional heterogeneity of the satellite glia remains to be determined. **d.** RT-qPCR for *Gfap* demonstrates that purified preparations of geniculate ganglion neurons (similar to **b**) contain $\approx 10^5$ times less *Gfap* mRNA than intact geniculate ganglia (similar to **a, c**). *Gfap* mRNA levels are normalized to *Snap25* mRNA which is expressed in all neurons, and relative levels were estimated by ΔΔCt (mean ± s.e.m. for three independent RNA samples prepared from separately isolated ganglia or separately prepared cell suspensions). *Gfap* was not detected among transcripts of any of the sequenced 96 geniculate ganglion neurons. Scale bars, 25μm.

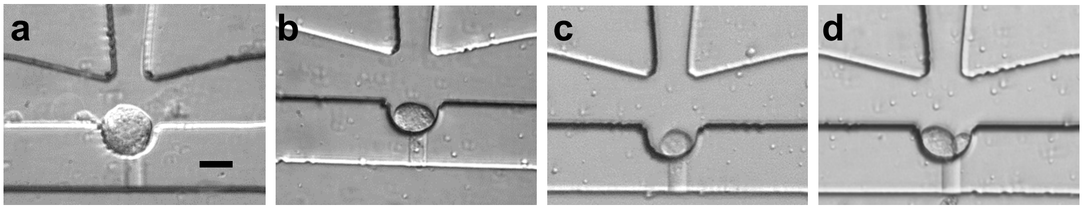

e

	Capture 1	Capture 2
Total sites on chip	96	96
Empty sites	3	7
Sites with multiple or unhealthy cells	32	36
Sites with single, healthy neurons	61	53
including small/medium neurons	57	46
including large/medium neurons	4	7
Single neurons selected for sequencing	48	48

f

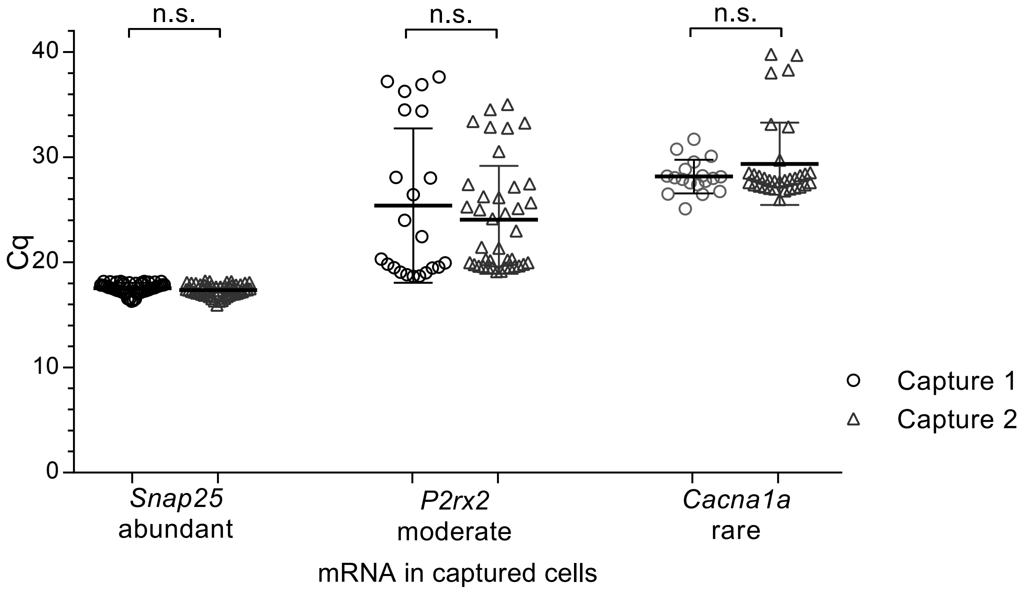

Supplementary Figure 2. Supplemental Figure S2. Captured geniculate ganglion neurons. A suspension of dissociated geniculate ganglia was enriched for neurons (see Methods and Supplemental Figure S1). The suspension was run through a Fluidigm IFC to capture cells. **a-c.** Examples of a large (**a**, ≈40μm), a medium (**b**, ≈25μm) and a small (**c**, ≈20μm) diameter neuron captured in IFC wells. **d.** Capture sites that included two cells were rejected for sequencing. Scale bar, 25μm. **e.** Two separate IFCs (96 capture sites each) were used. Of these, 48 singly-captured neurons were selected from each IFC for sequencing. The table shows the characteristics of cells at all 96 capture sites on each IFC. **f.** Quality control qPCRs for mRNA, cDNA synthesis and pre-amplification of 96 single cells selected for sequencing, plotted separately for the two IFCs. *Snap25, P2rx2* and *Cacna1a*, expressed at abundant, moderate, or low copy, respectively, were assessed for each neuron selected for sequencing. For each mRNA tested, the distribution across the two captures was not significantly different (unpaired t-test); plot displays mean ± s.d. Note, however, the broad range of Cq values for *P2rx2*) spanning ≥50,000 ($2*10^{18}$) fold differences.

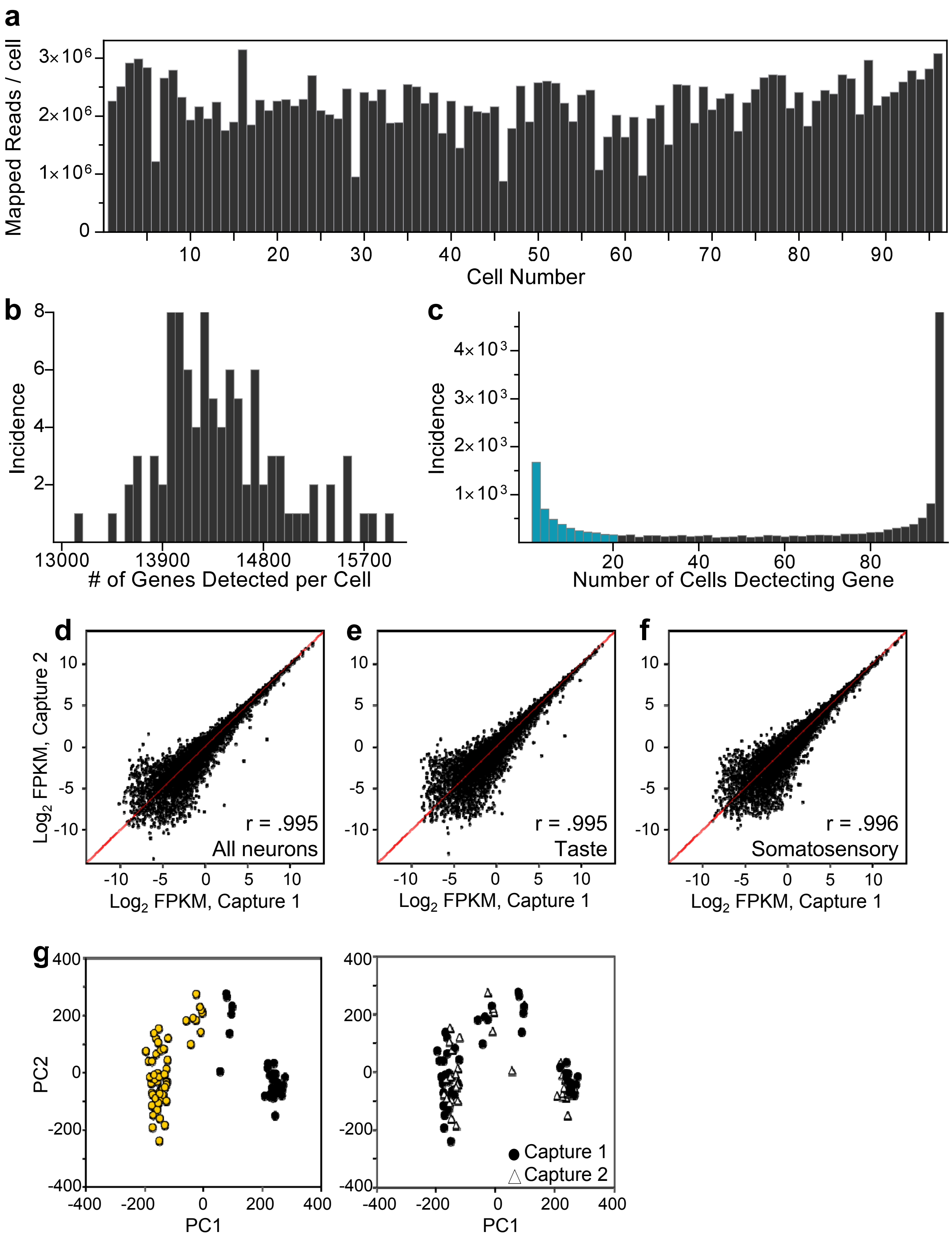

Supplementary Figure 3. Characteristics of single cell sequencing data and comparison of two capture preparations. 48 single isolated single cells from each of two IFCs were selected for sequencing (Methods). To confirm that the two preparations did not introduce a systematic bias, we evaluated the sequence data in several ways as detailed below. **a.** Total number of raw sequencing reads obtained for each of 96 cells are plotted. Capture 1 neurons: mean ± s.d. $2.47*10^6 \pm 0.42*10^6$ reads/cell; Capture 2 neurons: $2.62*10^6 \pm 0.57*10^6$ reads/cell. The two populations of 48 cells from each capture were not

significantly different (p=0.142 Student's t-test, 2-tailed). **b.** Histogram of number of genes detected per cell (Mean ± s.d. 14,426 ± 539) demonstrates that all 96 cells yielded adequate sequencing depth. **c.** Histogram of the number of cells in which a given gene was detected, for each of $\approx 1.7*10^4$ genes. Of these, 6773 genes were expressed in over 95% of all cells. Conversely, 4451 genes were expressed in ≤ 20 cells (blue bars) and are likely to include genes representing distinct cell types. **d.** Scatter diagram showing average FPKM of each gene from Capture 1 plotted against average in Capture 2 (n= 48 neurons in each capture). **e.** Scatter plot comparing average FPKM of all genes in 28 vs 31 *taste* neurons in the two captures (as identified in Figure 2). **f.** Scatter plot comparing average FPKM of all genes in 20 vs. 17 *somatosensory* neurons in the two captures. In each matrix (**d**, **e**, **f**) the red diagonal indicates perfect correlation (r=1); data points yield correlation coefficients of ≥0.995, indicating cells in the two captures are highly similar. **g.** PCA for all cells and all genes from Main Figure 1b is reproduced at left (yellow are taste neurons, black are somatosensory) and re-plotted at right to display neurons from the two captures using different symbols (Δ, ●). At right, each grouping includes cells from both preparations, confirming the similarity of the two preparations.

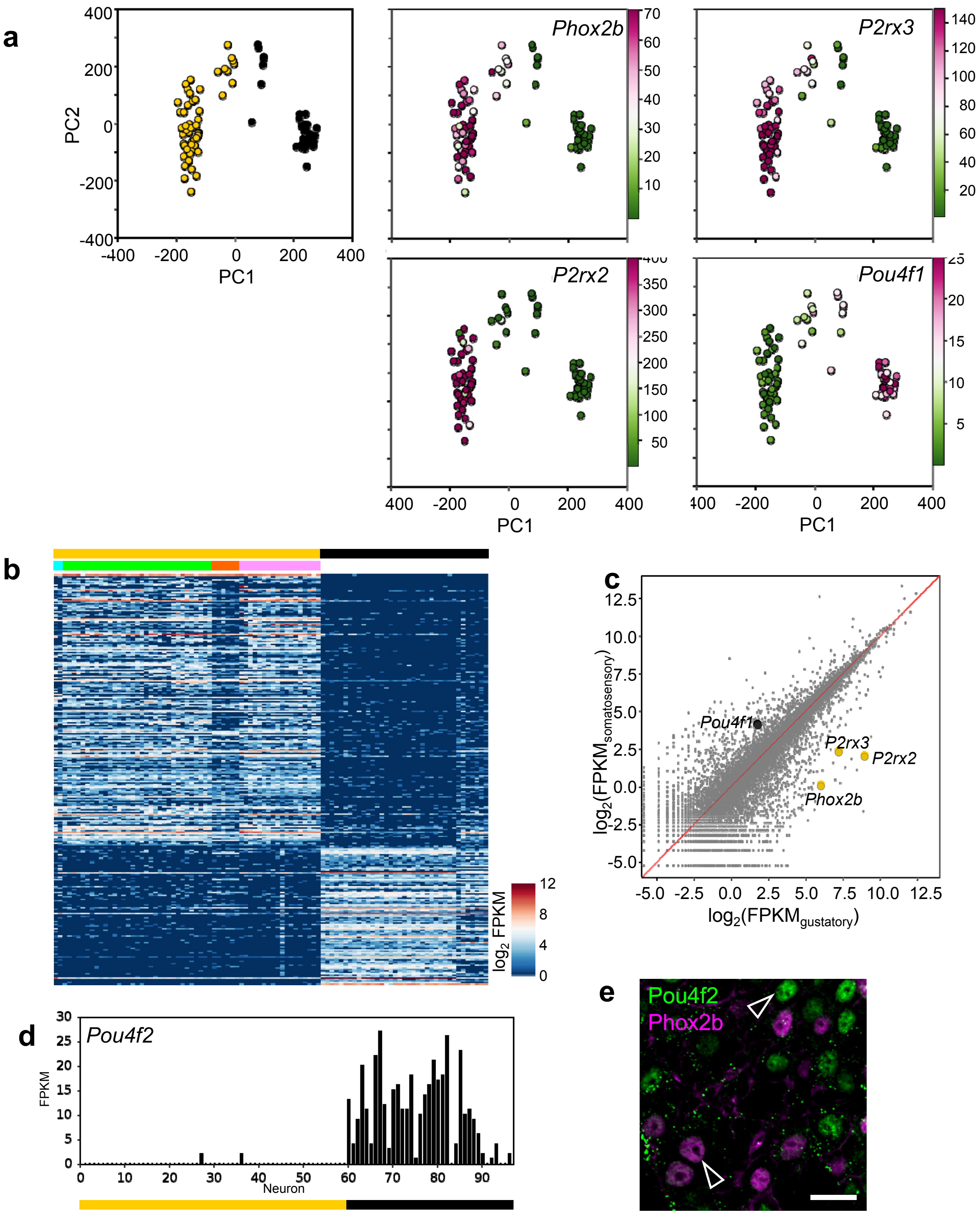

Supplementary Figure 4. Expression of taste and somatosensory markers in geniculate ganglion neurons. a. PCA of all genes expressed in all 96 neurons is reproduced from Figure 1b,and is used as a template to display FPKM values of taste-expressed genes, *Phox2b*, *P2rx3*, and *P2rx2*, and a previously reported determinant of somatosensory neurons, *Pou4f1*. The expression level of each gene (FPKM value) in each of the 96 cells is represented according to the adjacent color scale. For instance, *P2rx3* is expressed at high or moderate levels in the left cluster, is essentially not-expressed in the right cluster, and is at low and variable levels in the cells that fall between the two main clusters. **b.** Heatmap of differentially

expressed genes (FPKM ≥10-fold higher in gustatory or in somatosensory neurons) that displayed average FPKM ≥4 in at least one of these groupings. 294 genes met these criteria and are shown on the heat map, sorted from most taste-selective at the top to most somatosensory-selective at the bottom. Cells are arrayed with 59 taste neurons on the left and 37 somatosensory neurons on the right. **c.** Scatter plot depicts ≈17,100 genes according to average FPKM value of each gene in 37 somatosensory (y-axis) versus 59 gustatory (x-axis) neurons. *Phox2b*, *P2rx3*, and *P2rx2* are indicated in yellow; *Pou4f1* is in black. **d.** FPKM values for *Pou4f2* across all 96 sequenced cells shows this gene is expressed almost exclusively in somatosensory (black) neurons. **e.** Double immunofluorescence for Phox2b and Pou4f2 shows that Pou4f2-immunoreactive nuclei are distinct from Phox2b-immmunoreactive (i.e. gustatory neuron) nuclei (Δ).

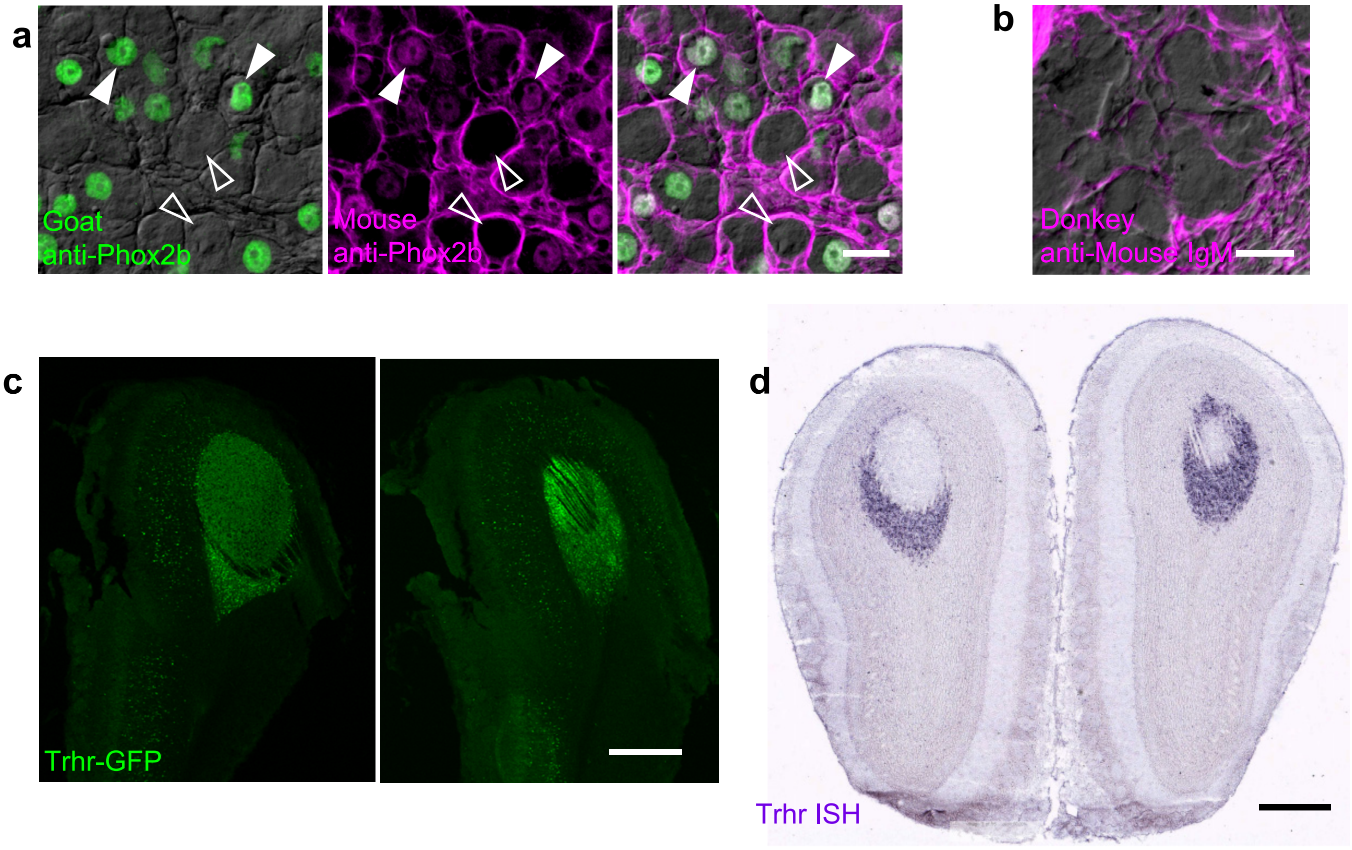

Supplementary Figure 5. Validation for anti-Phox2b antibody (a,b) and *Trhr*-GFP transgenic mouse (c,d). a. Two independent antibodies against Phox2b label the same neurons in geniculate ganglion.Cryosections were stained with goat anti-Phox2b and a mouse monoclonal anti-Phox2b and then visualized with secondary antibodies. Some nuclei (▲) stained with both antibodies while other nuclei (Δ) stained with neither. The reticular pattern around neurons derives from the anti-mouse secondary antibody (i.e. background staining).. **b.** Even in the absence of a primary antibody, the anti-mouse IgG secondary antibody employed in **a.** gives a non-specific reticular pattern signal in mouse ganglia. However, nuclei are unstained, demonstrating that nuclear staining in **a** is attributable to mouse anti-Phox2b primary antibody. **c.** Confocal images of two coronal sections of an olfactory bulb from an adult *Trhr*-GFP mouse, immunostained with chicken anti-GFP. GFP is prominently expressed in the accessory olfactory bulb. **d.** The pattern of GFP expression in the *Trhr*-GFP mouse in **c.** is very similar to the distribution of endogenous *Trhr* mRNA, visualized by *in situ* hybridization in an adult (P56) mouse, as presented in Allen Brain Atlas (http://mouse.brain-map.org/gene/show/21802; Trhr-RP_Baylor_103086–coronal. Image credit: Allen Institute).Scale bars, 20μm (a-c) or 500μm (d,e).

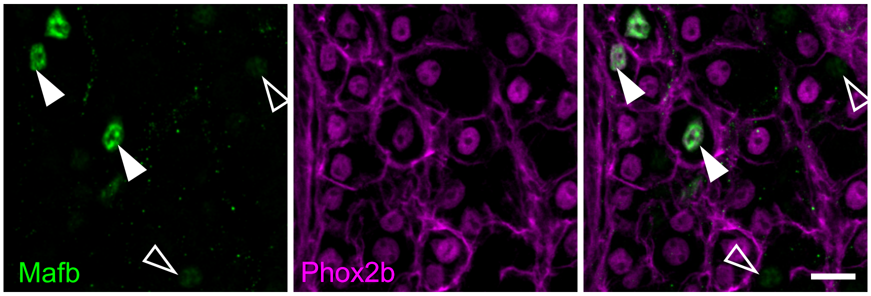

Supplementary Figure 6. Mafb+ neurons are taste neurons. We showed that Mafb-immunoreactive nuclei in geniculate ganglia do not express Prrxl1 (Figure 5d) and hence, are not somatosensory. Here, we show that mouse geniculate ganglion neurons that stain brightly with Mafb also express Phox2b (▲). Neurons staining faintly for Mafb are not Phox2b+ (Δ) and are likely to be somatosensory neurons, as predicted from the FPKM values and immunoreactivity with anti-Prrxl1 (Figure 5b,d,i). The mouse anti-Phox2b used here is the same one shown in Supplemental Figure S5a,b. The reticular staining around neurons is background immunostaining derived from the anti-mouse secondary antibody. In sections from 3 mice, all 25 Mafb-bright nuclei were Phox2b+ and accounted for 7% of Phox2b+ neurons, similar to their representation among the 96 sequenced neurons.

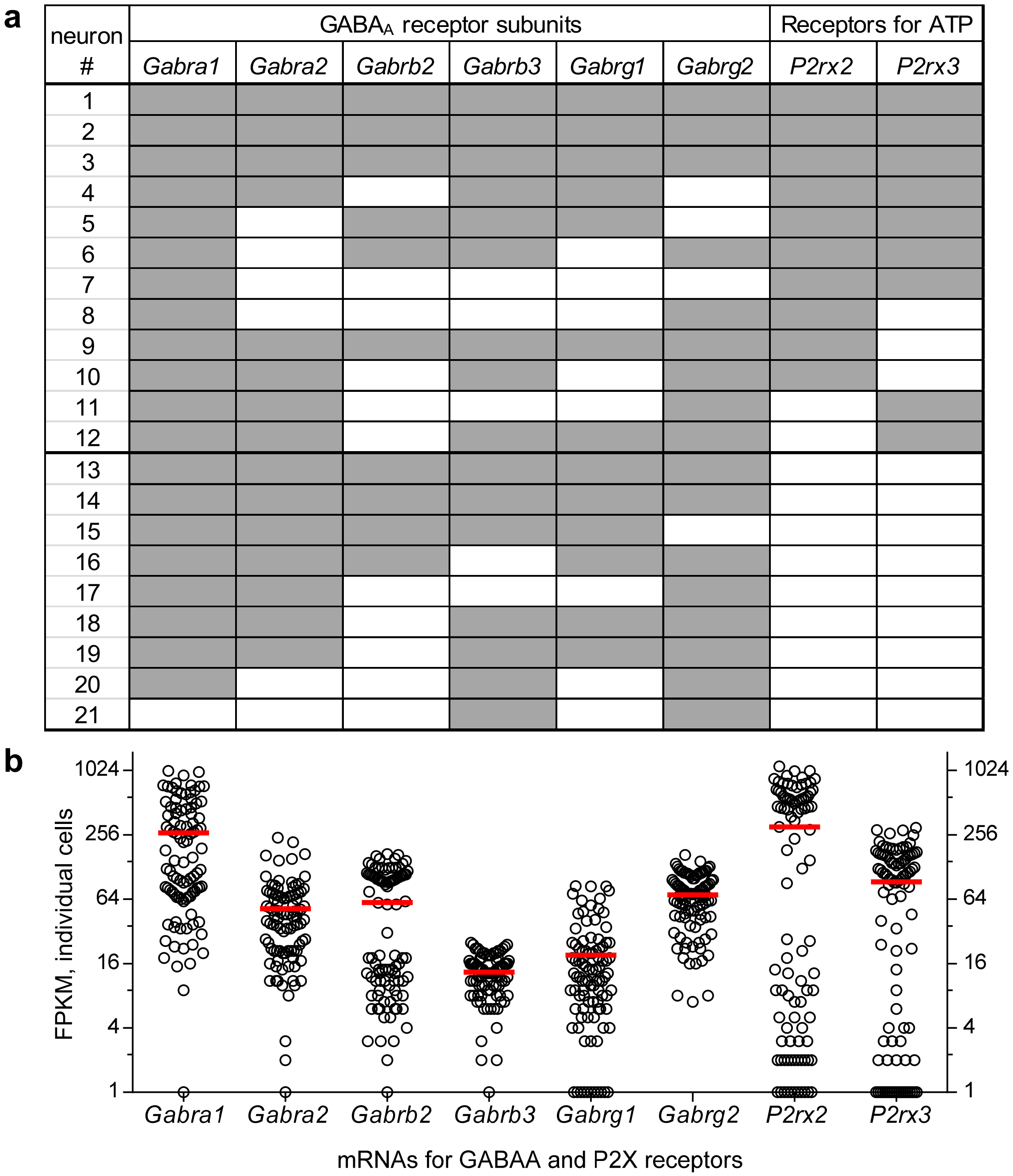

Supplementary Figure 7. Expression of GABAA receptor subunits in individual geniculate ganglion neurons – comparing RT-PCR and sequencing data. a. cDNA was synthesized from 21 single neurons, captured manually. Approx. 5% of each single-cell cDNA was used to check quality by PCR for Snap25. The remaining 95% was divided equally among PCRs for six subunits of GABAA and two subunits of P2X receptors. The presence (grey) or absence (clear) of specific product is indicated for each cell and each reaction in the table. **b.** The FPKM values for the same genes for all 96 cells sequenced are shown along with the mean (red) for each gene. Genes with high FPKM across all cells (such as *Gabra1*) are also detected in nearly every cell by single-cell PCR in **a**. Genes that display highly variable FPKM values (such as P2rx2, P2rx3) are detected by single-cell PCR in proportionately fewer cells.

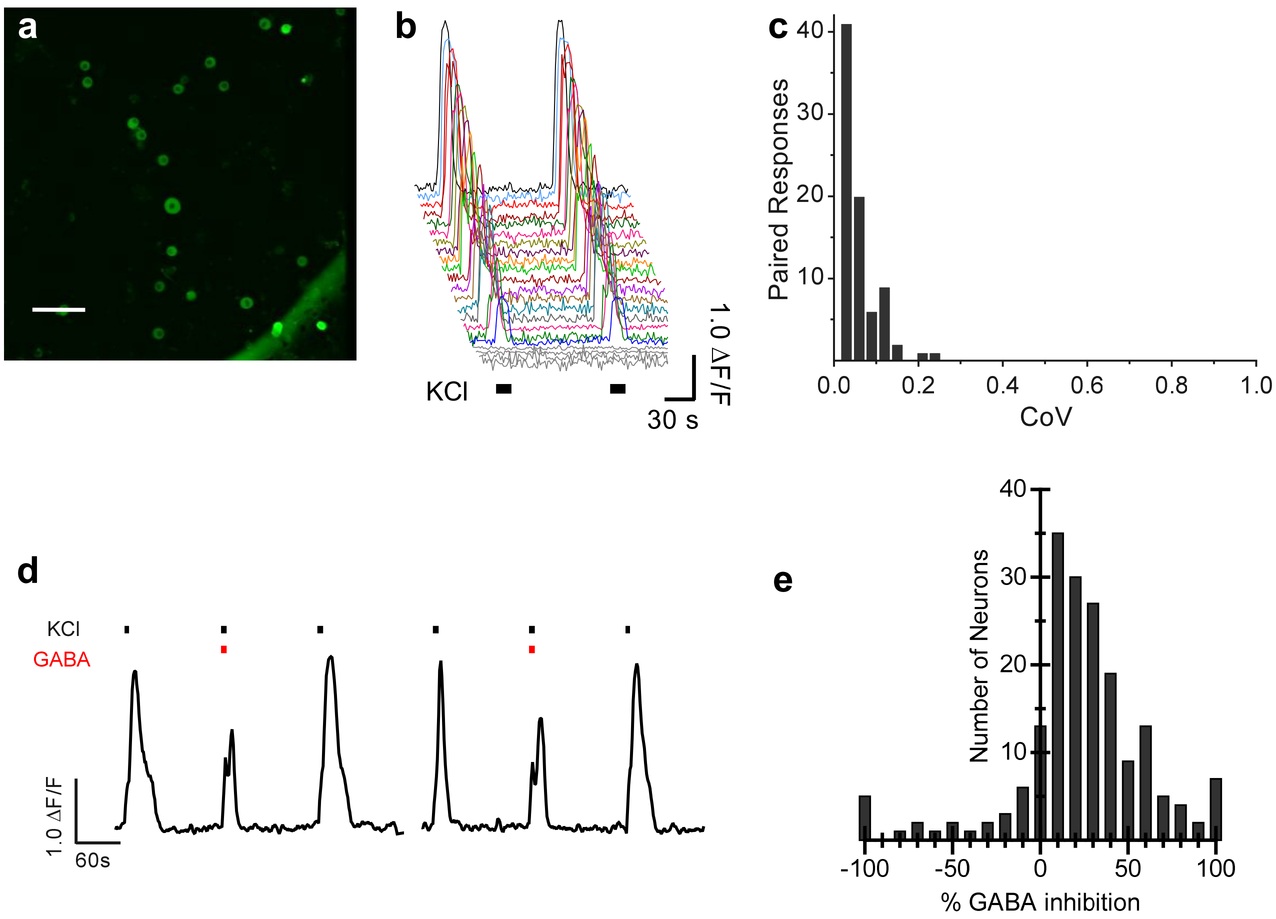

Supplementary Figure 8. Ca^{2+} responses in dissociated geniculate ganglion neurons and measuring responses to GABA. a. Dissociated geniculate ganglion preparation from *Pirt*-GCaMP3 mice prior to Ca^{2+} imaging. GCaMP is expressed only in neurons and in all neurons. Scale bar, 100μm. **b.** Calcium response profiles of all 23 neurons in **a.** when 30mM KCl was applied for 9 seconds (black bars). **c.** In two preliminary experiments, we confirmed the stability of the preparation of GCaMP3-expressing neurons by measuring 2 successive responses to KCl (as in **b.**) and another pair of KCl responses 6-10 min later. We calculated the coefficient of variation (σ/μ, CoV) for each pair of responses for each of 40 neurons. The average CoV was 0.03 and 90% of the neurons showed CoV ≤ 0.11. The highly consistent responses to KCl permitted us to quantify the effect of GABA in subsequent experiments. **d.** Representative traces of a neuron responding twice sequentially to 30mM KCl, in the absence or presence of 100μM GABA. **e.** Histogram of the effect of GABA on KCl-elicited Ca^{2+} responses in 187 neurons. The majority (80%) of neurons showed a 10-100% inhibition of the Ca^{2+} response when KCl and GABA were co-applied.

Protein / *gene*	Accession #	Forward Primer (5'→3')	Reverse Primer (5'→3')	Product, bp	Annl °C
SNAP25 /*Snap25*	NM_011428	ggcaataatcaggatggagtag	agatttaaccacttcccagca	310	58
P2X2 / *P2rx2*	NM_153400	caccaccactcgaactctca	ggtacgcaccttgtcgaact	217	58
P2X3 / *P2rx3*	NM_145526	caaagccaggaagtttgagg	gttctgcagcccaaggataa	179	58
5HT3a /*Htr3a*	NM_013561	ggcacctggtcctagacaga	caagtcctgaggtcctccaa	170	58
GABA-A α1 / *Gabra1*	NM_010250	ccaagtctccttctggctca	cggttctatggtcgcacttt	261	59
GABA-A α2 / *Gabra2*	NM_008066	ttgggacgggaagagtgtag	catcctgtcgattttgctga	232	59
GABA-A β2 / *Gabrb2*	NM_008070	tatggcccttctggaatacg	cccattactgcttcggatgt	204	59
GABA-A β3 / *Gabrb3*	NM_008071	gaagaagcttgcggagaaga	tggcattcacatcggttaga	326	59
GABA-A γ1 / *Gabrg1*	NM_010252	aggcaggaagctgaaaaaca	gcaaaagctgttgggaaaaa	255	59
GABA-A γ2 / *Gabrg2*	NM_177408	atcaatggaagcgcagttct	taggagaccttgggcagaga	334	59
α1A/*Cacna1a*	NM_007578	ctctgggccgatacactgat	gggatgatgatgatggtggt	164	58
Phox2b / *Phox2b*	NM_008888	tgagacgcactaccctgaca	tcagtgctcttggcctcttt	219	60
Drg11 / *Prrxl1*	NM_001001796	gcccaaacacactacccaga	cctccatttggctcttcggt	111	58
GFAP / *Gfap*	NM_010277	gcactcaatacgaggcagtg	gctctagggactcgttcgtg	207	60

Supplementary Table 1. Oligonucleotide primers used for RT-qPCR.

Primary Antibodies						
**Antigen**	**Antibody source, catalog #, Lot #**			**Host**	**Dilution**	**Validation**
Calb1	Neuromics	CH22118	401976	Chicken	1:1000	Concordant patterns by immunostaining and RNAseq for somata of geniculate ganglion neurons (Figure 8b,d)
Foxg1	Abcam	ab18259	GR252396-1	Rabbit	1:5000	Loss of staining in KO tissue [1]
Gabra1	Millipore	06-868	1975358	Rabbit	1:1000	Immunohistochemical staining matches zygosity[2]
GFP	Aves Labs	GFP-1020	0511FP12	Chicken	1:3000	Lack of staining on non-transgenic tissues (in-lab)
Mafb	Santa Cruz	sc-10022	L2115	Goat	1:500	Concordance of immunohistochemical staining and *in situ* hybrization[3]
NeuN-Al488	Millipore	MAB377X	2061436	Mouse	1:1000	Staining on only neuronal nuclei in ganglia
P2rx2	Alomone	APR003	AN1050	Rabbit	1:1000	Loss of staining in KO tissue[4]
Phox2b	Santa Cruz	sc-13224	C1016	Goat	1:500	Two independent antibodies stain the same nuclei (Supplementary Figure 5a).
Phox2b	Santa Cruz	sc376997	H1313	Mouse	1:200	
Pou4f2	Santa Cruz	sc-31989	I0513	Goat	1:200	Concordance of immunostaining with retinal ganglion cell types [5]
Prrxl1	Deolinda Lima, University of Porto			Rabbit	1:1000	Loss of staining in KO tissue [6]

Secondary Antibodies						
**Antigen**	**Antibody Source**	**catalog #**	**Lot #**	**Host**	**Fluor**	**Working concentration**
Rabbit IgG	ThermoFisher	A21207	1602780	Donkey	Alexa 594	1:1000
Rabbit IgG	ThermoFisher	A31573	1182665	Donkey	Alexa 647	1:1000
Goat IgG	ThermoFisher	A11055	1687906	Donkey	Alexa 488	1:1000
Goat IgG	ThermoFisher	A11058	71427	Donkey	Alexa 594	1:1000
Goat IgG	ThermoFisher	A21447	1010075	Donkey	Alexa 647	1:1000
Mouse IgG	Jackson Immunoresearch	715-585-150	124676	Donkey	Alexa 594	1:1000
Chicken IgY	Jackson Immunoresearch	703-545-155	126213	Donkey	Alexa 488	1:1000

1. Tian C *et al* (2012) Foxg1 has an essential role in postnatal development of the dentate gyrus. *J Neurosci* 32:2931-49.
2. Zhou C *et al* (2013) Altered cortical GABAA receptor composition, physiology, and endocytosis in a mouse model of a human genetic absence epilepsy syndrome. *J Biol Chem* 288:21458-72.
3. Howell DM *et al* (2007) Molecular guidance cues necessary for axon pathfinding from the ventral cochlear nucleus. *J Comp Neurol* 504:533-49.
4. Housley GD *et al* (2013) ATP-gated ion channels mediate adaptation to elevated sound levels. *Proc Natl Acad Sci USA* 110:7494-9.
5. Xu HP *et al* (2015) Spatial pattern of spontaneous retinal waves instructs retinotopic map refinement more than activity frequency. *Dev Neurobiol* 75:621-40.
6. Rebelo S *et al* (2007) DRG11 immunohistochemical expression during embryonic development in the mouse. *Dev Dyn* 236:2653-60.

Supplementary Table 2. Antibodies used and their validation.
